# High frequency of multidrug-resistant (MDR) *Klebsiella pneumoniae* harboring several β-lactamase and integron genes collected from several hospitals in the north of Iran

**DOI:** 10.1186/s12941-021-00476-1

**Published:** 2021-09-28

**Authors:** Mojgan Farhadi, Mohammad Ahanjan, Hamid Reza Goli, Mohammad Reza Haghshenas, Mehrdad Gholami

**Affiliations:** 1 Department of Microbiology and Virology, Faculty of Medicine, Mazandaran University of Medical Sciences, Sari, Iran; 2 Antimicrobial Resistance Research Center, Communicable Diseases Institute, Mazandaran University of Medical Sciences, Sari, Iran

**Keywords:** *K. pneumoniae*, Antibiotic resistance, Integron, β-lactamase, MDR

## Abstract

**Background:**

*Klebsiella pneumoniae* is one of the leading causes of hospital outbreaks worldwide. Also, antibiotic-resistant *K. pneumoniae* is progressively being involved in invasive infections with high morbidity and mortality. The aim of the current study was to determine antimicrobial susceptibility patterns and the incidence of resistance genes (integron types and β-lactamase-encoded genes) among clinical isolates of *K. pneumoniae*.

**Methods:**

In this cross-sectional study, a total of 100 clinical samples were obtained from hospitalized patients in three teaching hospitals in the north of Iran, from November 2018 and October 2019. Antimicrobial susceptibility testing was performed using disk agar diffusion test in line with CLSI recommendations. For colistin, minimum inhibitory concentration (MIC) was determined using broth microdilution. Based on antibiogram, multi-drug resistant (MDR) and extensive-drug resistant (XDR) strains were detected. Finally, integron types and β-lactamase resistance genes were identified using polymerase chain reaction technique.

**Results:**

The most and least clinical samples were related to the urine and bronchoalveolar lavage, respectively. Based on the antibiogram results, amikacin and gentamicin exhibited good activity against *K. pneumoniae* strains in vitro. The high resistance rate (93%) to ampicillin/sulbactam predicts the limited efficacy of this antibiotic, in the hospitals studied. Among all the 100 isolates, the frequency of MDR and XDR phenotypes were 58% and 13%, respectively, while no pan-drug resistant (PDR) strains were found. In the MDR *K. pneumoniae* strains, the prevalence of *bla*_SHV_*, bla*_TEM_*, bla*_CTX-M-15_*, bla*_KPC_*, bla*_OXA-48_*, bla*_NDM_ β-lactamase genes were 91.4%, 82.7%, 79.3%, 29.3%, 36.2% and 6.9%, respectively, however 91.4% of the isolates were carrying *intI* gene. Class II and III integrons were not detected in any isolates.

**Conclusion:**

The MDR *K. pneumoniae* is becoming a serious problem in hospitals, with many strains developing resistance to most available antimicrobials. Our results indicate co-presence of a series of β-lactamase and integron types on the MDR strains recovered from hospitalized patients. The increasing rate of these isolates emphasizes the importance of choosing an appropriate antimicrobial regimen based on antibiotic susceptibility pattern.

**Supplementary Information:**

The online version contains supplementary material available at 10.1186/s12941-021-00476-1.

## Background

Among the *Klebsiella* spp, *Klebsiella pneumoniae (K. pneumoniae* or KP) a gram- negative encapsulated bacterium, is responsible for up to 10% of nosocomial infections [1]. This organism causes a wide range of infections, such as pneumonia, burn and urinary tract infections (UTIs), septicemia, and meningitis [2]. Management of the infections caused by antibiotic-resistant *K. pneumoniae* is problematic due to the bacterium’s intrinsic and acquired resistance to a broad spectrum of the drugs, such as β-lactams. MDR strains can be fairly challenging to treat, especially for elderly, immunosuppressed individuals, or infants with immature immunity [3]. The β-lactamase-producing *K. pneumoniae* can destroy a varied range of β-lactams such as penicillins, carbapenems, and cephalosporins [4]. The key mechanisms of resistance *K. pneumoniae* uses against these antimicrobials are hyperexpression of chromosomal cephalosporinases and production of plasmid-encoded Ambler class A [Extended spectrum β-lactamases (ESBLs)], B (Metallo-β-lactamases) and D (oxacillinases) β-lactamases [5]. ESBLs are plasmid-borne enzymes that hydrolyze the oxyimino β-lactam ring found in 3rd generation cephalosporins and aztreonam. The extensive use of numerous β-lactam agents in recent decades has led to the appearance of ESBLs, which are frequently derivatives of TEM-1 and SHV-1 enzymes [6]. Carbapenems are the β-lactams of choice for the treatment of infections caused by ESBL-producing *K. pneumoniae*. Ambler class B enzymes which play a critical role in drug resistance against carbapenems, are zinc dependent and inhibited by EDTA [7]. Resistance genes have a high ability to spread, because the genes have commonly found on the transferable elements such as integrons, insertion sequences (IS) and transposons [8, 9]. Integrons, a segment of double-strand DNA sequence, are immobilized, but contain an integrase (*intI*)-encoding gene that allows the insertion of the resistance gene cassettes between highly conserved nucleotide sequences. Although several types of integrons have been identified, class I, II and III integrons are the most common types in the clinical settings [10, 11]. In recent years, MDR *K. pneumoniae* strains producing ESBLs-, MBLs, and KPC resistance genes have been progressively found in many regions of Iran [12–14]. Despite the high importance of this issue, only a restricted number of reports have originated from the north of Iran addressing the frequency and co-existence of resistance genes among the clinical isolates of *K. pneumoniae*. Therefore, this study was performed to determine the antibiotic resistance profiles, incidence of MDR, XDR and PDR phenotypes and also prevalence of β-lactamase and integron resistance genes among *K. pneumoniae* strains isolated from hospitalized patients in the north of Iran.

## Methods

### Study design and sampling

**Fig. 1:**
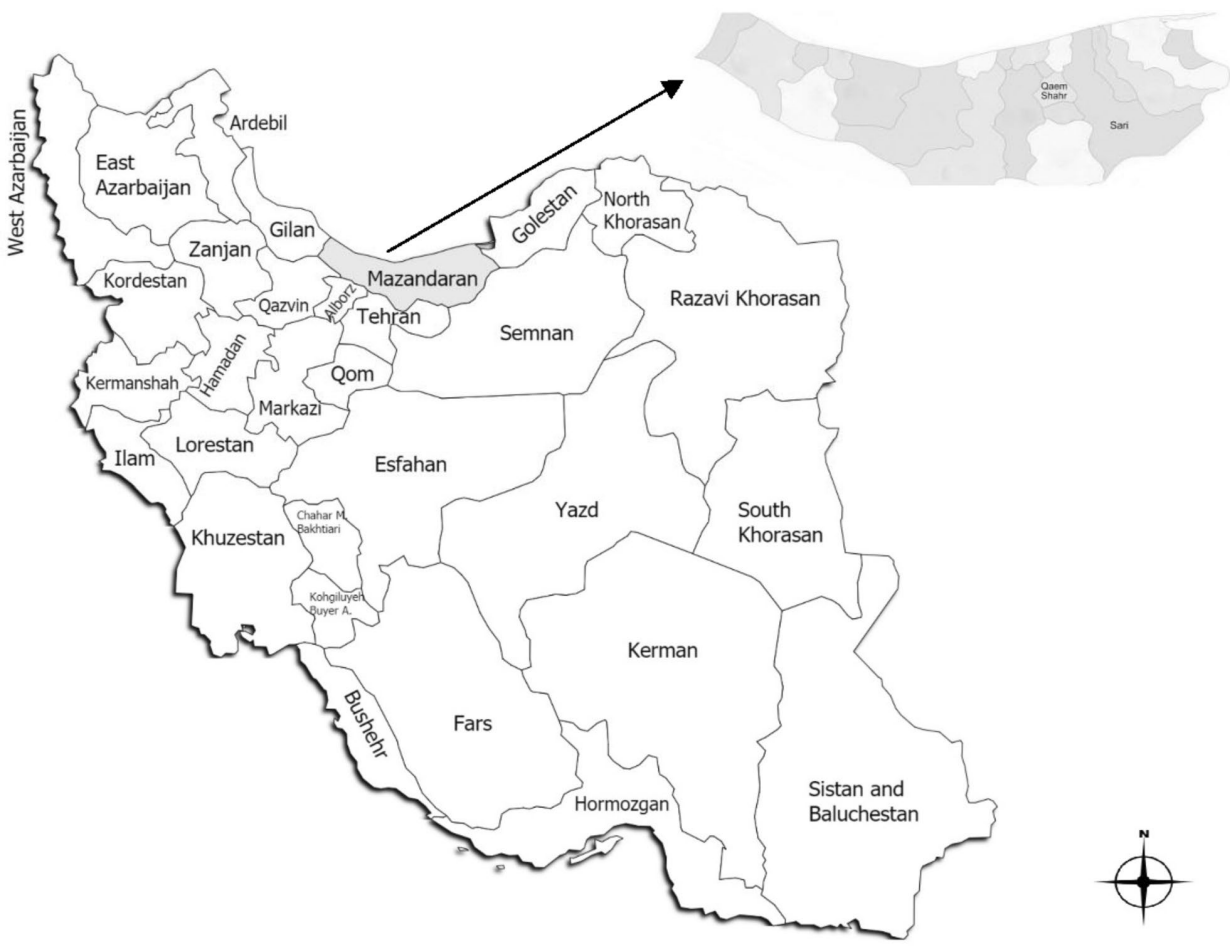
Map of Iran displaying the study province (gray zone) (Mazandaran province, northern Iran), with the geographical location of the collection centers in the cities of Sari and Qaem Shahr

In this descriptive cross-sectional study, based on the previous study [15], using the following formula n = z^2^P (1 − P)/d^2^, on a total of 100 non-duplicated samples, which were obtained from the one-year from November 2018 to October 2019. The clinical samples were collected from hospitalized patients of three educational hospitals in Mazandaran province. 1: Razi Teaching Hospital located in Qaem Shahr (Qaem Shahr is a city in Mazandaran province, in northern Iran) 2: Imam Khomeini Teaching Hospital located in Sari (Sari is the largest and the capital city of Mazandaran province, in northern Iran) and Zare Teaching Hospital located in Sari. These hospitals currently operate under the Mazandaran University of Medical Sciences (MAZUMS). The geographic region of the studied hospitals is shown in Fig. 1. One hundred *K. pneumoniae* isolates were recovered from different clinical sources including blood, sputum, bronchoalveolar lavage (BAL), wound exudates, urine, cerebrospinal fluid (CSF) and synovial fluid. In this study, all clinical specimens from any human source which contain *K. pneumoniae*, including both gender, and from all age groups including infants to elderly were included. Hence, *K. pneumoniae* strains isolated from out-patients, as well as other species of *Klebsiella* and mixed and/or contaminated plates were excluded.

### Microbiological method

The samples were cultured on MacConkey agar (Merck, Germany), then incubated at 37 °C for 24 h. The recovered colonies were initially identified using conventional biochemical and microbiological tests, including motility (−), urease (+), oxidase (−), lysine decarboxylase (+), citrate utilization (+), Triple sugar iron agar (acid/acid), Hydrogen sulfide (-), Methyl Red-Voges Proskauer (−/+), and Indole (−) [16]. Further confirmation of *K. pneumoniae* strains at the species level was accomplished by the analytical profile index (API) 20E kit (bioMérieux, La-Balme-les-Grottes, France) according to the manufacturer’s instructions [17]. All isolates were preserved in the Luria–Bertani (LB) broth (Merck, Co., Germany) containing 20% glycerol at −80 °C for further use.

### Antimicrobial susceptibility testing (AST)

In concordance with the Clinical and Laboratory Standards Institute; CLSI. 2018 [18], antimicrobial susceptibility testing was done on the Mueller–Hinton agar plates (Merck Co., Germany) by disk agar diffusion (DD) method against 16 following antimicrobials: levofloxacin (LEV; 5 µg); ceftazidime (CAZ; 30 µg), cefotaxime (CTX; 30 µg), cefepime (FEP; 30 µg), ertapenem (ETP; 10 µg), amikacin (AK; 30 µg), meropenem (MER; 10 µg), ceftriaxone (CRO; 30 μg), ampicillin/sulbactam (SAM; 10/10 µg), cefoperazone (CFP; 75 µg), imipenem (IPM; 10 µg), nitrofurantoin (NIT;300 µg), gentamicin (GM; 10 µg), ciprofloxacin (CIP; 5 µg), tetracycline (TET; 30 µg), and trimethoprim-sulfamethoxazole (SXT; 1.25/23.75 µg) (MAST Diagnostics, Merseyside, UK). The MDR and possible XDR/PDR strains were recognized according to the guidelines suggested by the European Center for Disease Control and Prevention (ECDC) [19]. *E. coli* ATCC 25922 was used as a quality control (QC) organism. Also, colistin susceptibility assay was done for carbapenems-resistance isolates by broth microdilution method according to the European Committee on Antimicrobial Susceptibility Testing (EUCAST) breakpoints [20]. The *E. coli* NCTC 13846 (a colistin-resistant strain) was used as QC in colistin minimum inhibitory concentration (MIC) determination. The primary screening of extended-spectrum β-lactamases (ESBL)-producing pathogens was assess by the profile of AST. Strains that display reduced susceptibilities to CAZ and/or CTX were temporarily regarded as ESBL-producer, and finally confirmed as instructed by CLSI.

### Genomic DNA extraction

Total genomic DNA of every isolate was obtained from the colonies grown on the LB medium (Merck, Darmstadt, Germany) using a High Pure PCR Template Preparation Kit (Roche, Germany), based on the manufacturer’s instruction. The extracted DNAs were quantified by measuring the absorbance at A260/A280 with Nanodrop spectrophotometer (ND-1000; Thermo Scientific; Wilmington, DE, USA), to evaluate the extracted DNA purity. Purified DNA was kept at − 20 °C until further use.

### Molecular methods

PCR primers were designed for the detection of integrase genes (*intI*, *intII*, and *intIII*), and resistance elements such as *bla*_CTX-M-15_, *bla*_TEM_, *bla*_SHV_, *bla*_KPC_, *bla*_OXA-48_ and *bla*_NDM_ genes. The primer sequences used in this work are listed in Additional file 1: Table S1, as previously described. Amplification reactions were performed in a final volume of 25 μl, containing 0.8 μl of the extracted DNA (0.5 μg), 12.5 μl of 2 × Master Mix (Amplicon Co., Denmark), including 1 × PCR buffer, 1.5 mmol/l MgCl_2_, dNTPs at a concentration of 0.15 mmol/l each dNTP, 1.25 U of Taq DNA polymerase (Amplicon Co., Denmark), 0.7 μl of 0.8 μM of each primer, and nuclease free sterile water, up to 25 μl. The PCR mixture reaction was amplified in a Techne TC-512 thermal cycler (Eppendorf, Hamburg, Germany) as follows: initial denaturation at 95 ˚C for 1 min, 32 cycles of denaturation for 30 s at 94 °C, annealing for 30 s at 60 °C and extension for 1 min at 72 °C, and a final extension for 5 min at 72 °C. PCR amplicon products were subjected to electrophoresis in a 1.0% agarose gel, stained with Gel Red™ (Biotium, USA) and photographed with ultraviolet illumination (Bio-rad, Hercules, USA). Both positive and negative controls were included in each run. The positive PCR products were sequenced by Bioneer Company (Korea). The nucleotide sequences alignments were analyzed with running Basic Local Alignment Search Tool (BLAST) at National Center for Biotechnology Information (NCBI) database (http://blast.ncbi.nlm.nih.gov/Blast.cgi).

### Statistical analysis

After collection of the data, statistical analysis was performed with the IBM SPSS Statistics 20 (SPSS Inc., Chicago, IL, USA) and a *p-value* less than 0.05 was considered as statistically significant.

## Results

### Bacterial isolation

A total of 100 non-duplicated *K. pneumoniae* were obtained from blood (n = 15), sputum (n = 5), BAL (n = 2), wound exudates (n = 10), urine (n = 65) and CSF (n = 3). The frequency of isolates in hospital wards were as follows: Intensive Care Units (ICUs) (n = 27), burn (n = 19), dialysis (n = 17), hematology-oncology (n = 12), respiratory care (n = 9), internal medicine (n = 8), neonatal intensive care unit (NICU) (n = 5), and surgery (n = 3). The mean age of the patients was 51.7 years (ranging from 15 to 91 years), where 58% (n = 58) of the patients were female and 42% (n = 42) of them were male. In our studied therapeutic centers, the prevalence of *K. pneumoniae* was as follows: 41% in Imam Khomeini, 45% in Zare and 14% from Razi hospitals affiliated to the Mazandaran University of Medical sciences (north of Iran).

### Determination of antibiotic resistance pattern

Based on the acquired antibiotic resistance pattern, the highest and lowest resistance rate was related to SAM (93%) and AK (8%), respectively (Table 1). Also, 58% (58/100) of the isolates were resistant to three or more antimicrobials (MDR), and 13% (13/100) isolates were XDR. No PDR isolates were found. No non-MDR strains were resistant to AK. Altogether, the frequency of resistance genes among MDR strains was significantly higher than in non-MDR strains (p < 0.05). Overall, among 45 carbapenem-resistant *K. pneumoniae* (CRKP) strains, 28 cases were resistant to colistin antibiotic with a MIC > 2. Beside, ESBLs were phenotypically identified in 64 (64%) of the all tested isolates, molecular analysis exhibited that all strains had at least one ESBL gene.

**Table 1:** Antimicrobial susceptibility pattern among MDR and non-MDR *K. pneumoniae* isolates

Antimicrobial agents	No. (%) in MDR-isolates			No. (%) in Non-MDR isolates		
	S	I	R	S	I	R
LEV	36 (36%)	3 (3%)	19 (19%)	38 (38%)	0 (0%)	4 (4%)
CAZ	10 (10%)	7 (7%)	41 (41%)	32 (32%)	2 (2%)	8 (8%)
CTX	11 (11%)	8 (8%)	39 (39%)	29 (29%)	4 (4%)	9 (9%)
FEP	15 (15%)	5 (5%)	38 (38%)	36 (36%)	3 (3%)	3 (3%)
ETP	35 (35%)	3 (3%)	20 (20%)	38 (38%)	1 (1%)	3 (3%)
AK	45 (45%)	5 (5%)	8 (8%)	41 (41%)	1 (1%)	0 (0%)
MER	17 (17%)	2 (2%)	39 (39%)	39 (39%)	0 (0%)	3 (3%)
CRO	14 (14%)	6 (6%)	38 (38%)	35 (35%)	3 (3%)	4 (4%)
SAM	1 (1%)	1 (1%)	56 (56%)	0 (0%)	5 (5%)	37 (37%)
CFP	11 (11%)	4 (4%)	43 (43%)	32 (32%)	1 (1%)	9 (9%)
IPM	26 (26%)	2 (2%)	30 (30%)	39 (39%)	0 (0%)	3 (3%)
NIT	2 (2%)	3 (3%)	53 (53%)	25 (25%)	13 (13%)	4 (4%)
GM	40 (40%)	1 (1%)	17 (17%)	35 (35%)	1 (1%)	6 (6%)
CIP	31 (31%)	0 (0%)	27 (27%)	65 (65%)	0 (0%)	7 (7%)
TET	22 (22%)	17 (17%)	19 (19%)	33 (33%)	6 (6%)	3 (3%)
SXT	13 (13%)	4 (4%)	41 (41%)	31 (31%)	2 (2%)	9 (9%)

*MDR* multidrug resistant, *S* susceptible, *I* intermediate, *R* resistant, *LEV* levofloxacin, *CAZ* ceftazidime, *CTX* cefotaxime, *FEP* cefepime, *ETP* ertapenem, *AK* amikacin, *MER* meropenem, *CRO* ceftriaxone, *SAM* ampicillin/sulbactam, *CFP* cefoperazone, *IPM* imipenem, *NIT* nitrofurantoin, *GM* gentamicin, *CIP* ciprofloxacin, *TET* tetracycline, *SXT* trimethoprim-sulfamethoxazole

### Molecular detection of resistance genes

**Fig. 2:**
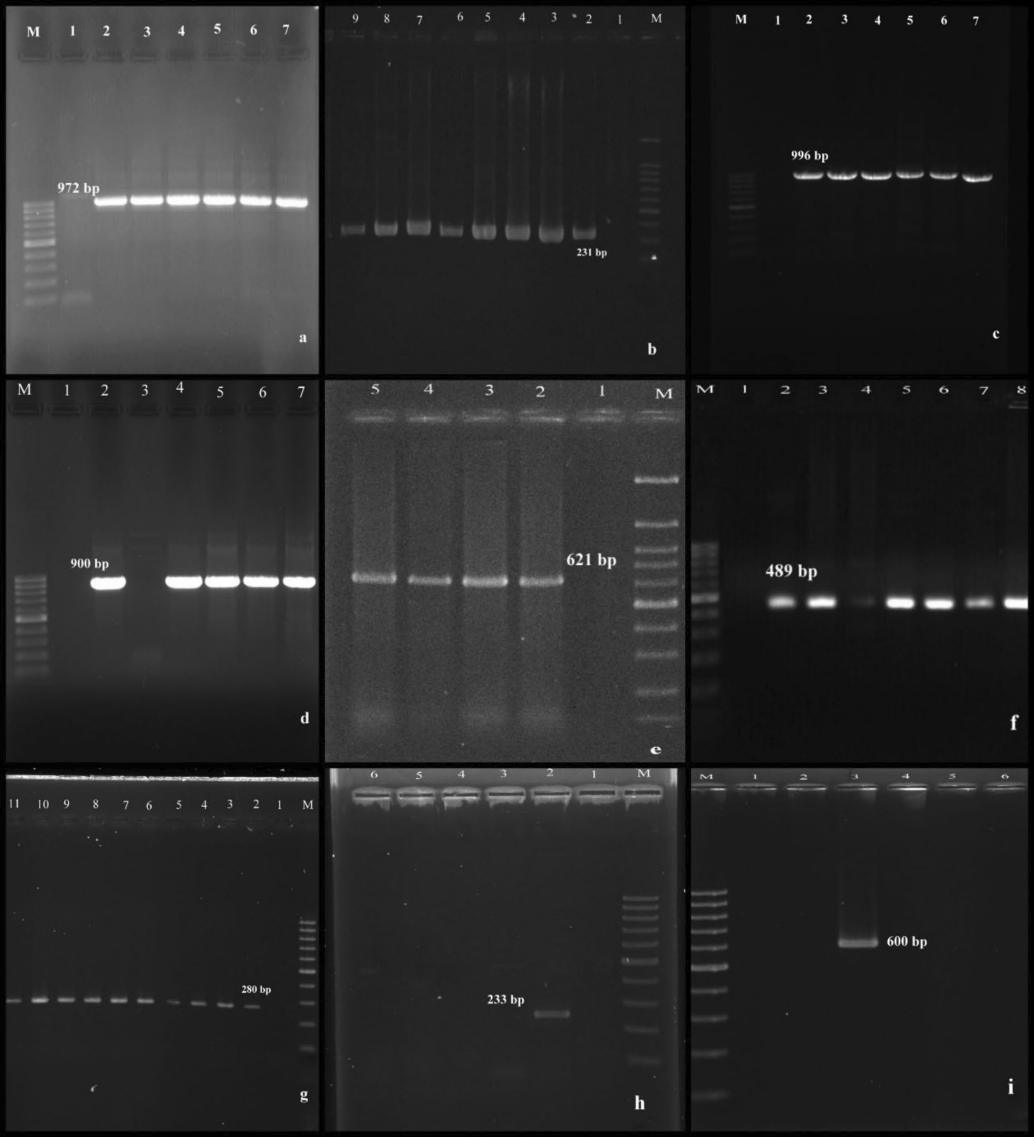
Agarose gel electrophoresis used for separation of the different PCR products. Lanes labeled “M-” correspond to ladder 100 bp. Lanes labeled “1” correspond to negative controls in multi-panel figures. Lanes labeled “2” correspond to positive controls in multi-panel figures; **a** amplified products of *bla*_TEM_ (972 bp), **b** *bla*_SHV_ (231 bp), **c** *bla*_CTX-M-15_: (996 bp), **d** *bla*_OXA-48_ (900 bp), **e** *bla*_NDM_ (621 bp), **f** *bla*_KPC_ (489 bp), **g** *intI* (280 bp), **h** *intII* (233 bp) and lane **i** *intIII* (600 bp)

Molecular analysis results showed that class I integrons were the predominant resistance transferable elements in our isolates. However, 91.4% and 11.9% of the MDR and non-MDR isolates were carrying *intI* gene, respectively. Class II and III integrons were not detected in any isolates. As shown in Table 2, the frequency of resistance genes in MDR isolates was higher than non-MDR. Among the MDR strains, the frequency of *bla*_SHV_*, bla*_TEM_*, bla*_CTX-M-15_*, bla*_KPC_*, bla*_OXA-48_*, bla*_NDM_ were 91.4%, 82.7%, 79.3%, 29.3%, 36.2% and 6.9%, respectively. The agarose gel electrophoresis of the PCR-amplified products for interest genes are shown in Fig. 2. Overall, the co-existence of the *bla*_SHV_*/bla*_TEM_, *bla*_TEM_*/bla*_CTX-M-15_, *bla*_SHV_*/bla*_CTX-M-15_, *bla*_CTX-M-15_*/bla*_OXA-48_, *bla*_SHV_*/bla*_OXA-48_, *bla*_TEM_*/bla*_OXA-48_, and *bla*_SHV_*/bla*_KPC_, were 21%, 18%, 13%, 8%, 5%, 4%, and 3%, correspondingly. Only one isolate was carrying *bla*_SHV_/*bla*_TEM_*/ bla*_CTX-M-15_/*bla*_KPC_/*bla*_OXA-48_ genes.

**Table 2:** Antimicrobial resistance genes and integron types in MDR and non-MDR *K. pneumoniae* isolates

Tested isolates	Resistance-encoding genes, n (%)						Integron types		
	*bla*_SHV_	*bla*_TEM_	*bla*_CTX-M-15_	*bla*_KPC_	*bla*_OXA-48_	*bla*_NDM_	*intI*	*intII*	*intIII*
MDR (n= 58)	53 (91.4%)	48 (82.7%)	46 (79.3%)	17 (29.3%)	21 (36.2%)	4 (6.9%)	53 (91.4%)	0 (0%)	0 (0%)
Non-MDR (n=42)	31 (73.8%)	18 (42.6%)	5 (11.9%)	3 (7.4%)	7 (16.6%)	0 (0%)	5 (11.9%)	0 (0%)	0 (0%)

## Discussion

The increasing rate of *K. pneumoniae* resistant strains against multiple antimicrobials is a major challenge in medical centers [30]. In a systematic review and meta-analysis article, Vaez et al., (2019), declared that there is a relatively high frequent antibiotic-resistant *K. pneumoniae* in Iran [31]. In the present study, the highest and lowest resistance rates were related to SAM (93%) and AK (8%), respectively. In this study, 33% of the isolates were considered as an IMP-resistant *K. pneumoniae* (IRKP); 30% of the MDR and 3% of the non-MDR strains, while no non-MDR strains were resistant to AK. In total, only eight isolates were resistant to AK; therefore, this antimicrobial was the best choice against the tested strains. The frequency of MDR and XDR isolates in our hospitals were 58% and 13%, respectively. In comparison with our data, Moghadas et al. reported that 7.5%, 16.1%, 32.9%, 34.1%, 36.4%, and 42.7% of their isolates were resistant to IPM, CIP, SXT, FEP, AN, and CAZ, respectively [32]. Another study also showed disparity with our data, where 89.5% of the *K. pneumoniae* strains were MDR [33], thus far higher than reported in the current work. Geographic distance, level of hygiene, type of specimens, date of study, sample size, and restriction on antibiotic usage may be the reasons for these inconsistencies.

In our study, colistin resistance was found in 62.22% CRKP strains, which we consider a surprisingly high level. Possibly the over-use of this antimicrobial during recent years in the treatment of infections caused by organisms resistant to less toxic antibiotics could be the cause of this phenomenon. In an earlier study from Iran, Farivar et al. reported that 16.9% of *K. pneumoniae* isolated from various clinical sources were resistant to colistin [34]. In another recent work performed in an Iranian teaching hospital involving 100 CRKP strains from hospitalized patients, 50% of them were resistant to colistin [35]. Previous reports from India, Kuwait, Turkey and United Arab Emirates revealed that the prevalence of colistin resistance among *K. pneumoniae* was 4%, 8%, 27.5%, and 31.4% [36–39]. Molecular analysis showed that the frequency of resistance genes in MDR isolates was higher than non-MDR ones. In our study, out of 58 MDR *K. pneumoniae*, *intI*, *bla*_SHV_, *bla*_TEM_, *bla*_CTX-M-15_, *bla*_OXA-48_, *bla*_KPC_, *bla*_NDM_ were detected in 91.4%, 91.4%, 82.7%, 79.3%, 36.2%, 29.3% and 6.9% of them by PCR. Mahmoudi et al. showed that out of 30 *K. pneumoniae* isolates, the frequency of *bla*_SHV_, *bla*_CTX-M-15_ and *bla*_TEM_ genes were 83% (n = 25), 70% (n = 21) and 57% (n = 17), respectively [40]. In another study, directed by Ghafourian et al., (2012), 36.1% (104/288), 7.6% (22/288) and 5.9% (17/288) of *K. pneumoniae* isolates in patients with urinary tract infection were positive for *bla*_SHV_, *bla*_CTX-M-15_ and *bla*_TEM_ genes, respectively [41]. Furthermore, 20% of the isolates were carrying *bla*_KPC_ gene.

Bina et al. reported that the resistance rates of *K. pneumoniae* isolates were 15.5%, 13.9%, 14.5%, 50%, 44.2%, 36.4%, 20.9%, 50%, 41.3%, 60.6% and 48.8% against ETP, IPM, MER, CTX, CAZ, FEP, cefoxitin, CRO, GM, piperacillin and aztreonam, respectively. Beside, their results indicated that the *bla*_KPC_ gene was not detected in any of the 41 CRKP isolates, while in phenotypic screening test for KPC, 80% of these isolates were positive. Regarding to these cases and restrictions mentioned about phenotypic methods, researchers rely on molecular-based techniques to confirm phenotypic results [42]. Also, the discrepancy could be due to at least one extended-spectrum cephalosporin and another mechanism such as an ESBL or AmpC-type enzyme with porin loss [43, 44]. An exciting point in our study is the low prevalence of *bla*_NDM_ gene. According to the findings of Fallah et al., the close distance of India and Pakistan to Iran, the large number of journeys between the countries, and the ease of resistance transfer among microorganisms have led us to think that it may be likely for antibiotic-resistant bacteria to have the same gene [45].

PCR test results showed that *intI* gene was detected in 58% of the isolates (53 MDR and 5 non-MDR). Concordance with our study, Derakhshan et al. showed that 25.8% (8/31) of their *K. pneumoniae* isolates were carried *intI*. In addition, they did not find class II and III integrons [46]. In contrast, Haddadi et al. (2019), found that the class I integron gene frequency among 54 MDR *K. pneumoniae* clinical strains was 37.6% [47]. Firoozeh et al. showed that 100% (150/150) and 36.7% (55/150) of their MDR *K. pneumoniae* carried *intI* and *intII* genes, respectively [48]. But this finding is contradictory to our reports. We did not find any class II and III integrons among all samples. This discrepancy could be due to differences in the source of samples, microbial genetic diversity and level of hygiene.

## Conclusion

This study has the potential to add to the body of literature regarding to MDR and/or XDR organisms in Iran. MDR *K. pneumoniae* is becoming a severe problem in hospitals, as many strains are developing resistance to most available antimicrobials. The increasing rate of these isolates emphasizes the importance of choosing an appropriate antimicrobial regimen based on antibiotic susceptibility patterns. The finding of the present study exposed a high prevalence of class I integron among MDR *K. pneumoniae* isolates and resistance genes especially *bla*_SHV_ and *bla*_TEM_ from Sari (north of Iran), which led to more attention to MDR strains.

## Supplementary Information

**Additional file 1: **Table S1: Primer sequences used for PCR amplification in this study and amplicon sizes.

## Data Availability

All data generated or analyzed during this work are included in this published article. Also the all data used to support the findings of this study are available from the corresponding author upon request.

## Abbreviations

BAL: Bronchoalveolar lavage
UTIs: Urinary tract infections
MDR: Multidrug-resistant
XDR: Extensively drug-resistant
DD: Disk agar diffusion
PCR: Polymerase chain reaction
CLSI: Clinical laboratory standards institute
QC: Quality control
CSF: Cerebrospinal fluid
BLAST: Basic local alignment search tool
NCBI: National Center for Biotechnology Information
EUCAST: European Committee on Antimicrobial Susceptibility Testing
LB: Luria–Bertani
ECDC: European Center for Disease Control and Prevention
ESBLs: Extended spectrum β-lactamases
IRKP: IMP-resistant *K. pneumonia*
CRKP: Carbapenem-resistant *K. pneumonia*
MBLs: Metallo-β-lactamases
KPC: *K. pneumoniae* Carbapenemase
NICU: Neonatal intensive care unit
ICUs: Intensive care units
