# Supplementary material for: High frequency of multidrug-resistant (MDR) Klebsiella pneumoniae harboring several β-lactamase and integron genes collected from several hospitals in the north of Iran
Source: Ann Clin Microbiol Antimicrob. 2021 Sep 28;20:70. doi: 10.1186/s12941-021-00476-1 (PMC8479884; doi:10.1186/s12941-021-00476-1)
Supplement: Supplementary file 1 — Additional file 1: Table S1: Primer sequences used for PCR amplification in this study and amplicon sizes. [file 12941_2021_476_MOESM1_ESM.docx]

Table S1: Primer sequences used for PCR amplification in this study and amplicon sizes

| Reference | Product size (bp) | Primer sequence (5'→3') | Target genes |
| --- | --- | --- | --- |
| 21 | 280 | F=5ʹ- CCTCCCGCACGATGATC-3ʹ  R=5ʹ- TCCACGCATCGTCAGGC-3ʹ | *intI* |
| 22 | 233 | F=5ʹ- TTATTGCTGGGATTAGGC-3ʹ  R =5ʹ- ACGGCTACCCTCTGTTATC-3ʹ | *int*II |
| 23 | 600 | F=5ʹ- AGTGGGTGGCGAATGAGTG-3ʹ  R =5ʹ- TGTTCTTGTATCGGCAGGTG-3ʹ | *intIII* |
| 24 | 996 | F=5ʹ- CACACGTGGAATTTAGGGACT-3ʹ  R =5ʹ- GCCGTCTAAGGCGATAAACA-3ʹ | *bla*_CTX-M-15_ |
| 25 | 972 | F=5ʹ- TCGGGGAAATGTGCGCG -3ʹ  R =5ʹ- TGCTTAATCAGTGAGGCACC -3ʹ | *bla*_TEM_ |
| 26 | 231 | F=5ʹ- AAGATCCACTATCGCCAGCAG-3ʹ  R=5ʹ- ATTCAGTTCCGTTTCCCAGCGG-3ʹ | *bla*_SHV_ |
| 27 | 489 | F=5ʹ- CTTGCTGCCGCTGTGCTG-3ʹ  R=5ʹ GCAGGTTCCGGTTTTGTCTC-3ʹ | *bla*_KPC_ |
| 28 | 900 | F=5ʹ -GGGGACGTTATGCGTGTATT-3 ʹ  R=5ʹ -GAGCACTTCTTTTGTGATGGC-3 ʹ | *bla*_OXA-48_ |
| 29 | 621 | F=5ʹ - GGTTTGGCGATCTGGTTTTC-3'  R=5ʹ - CGGAATGGCTCATCACGATC-3' | *bla*_NDM_ |
